# Use of Wheat Straw for Value-Added Product Xylanase by *Penicillium chrysogenum* Strain A3 DSM105774

**DOI:** 10.3390/jof7090696

**Published:** 2021-08-27

**Authors:** Amira A. Matrawy, Ahmed I. Khalil, Heba S. Marey, Amira M. Embaby

**Affiliations:** 1 Environmental Studies Department, Institute of Graduate Studies and Research, Alexandria University, Alexandria 21526, Egypt; amira.matrawy30@alexu.edu.eg (A.A.M.); airkhalil@gmail.com (A.I.K.); hebasaid06@gmail.com (H.S.M.); 2 Biotechnology Department, Institute of Graduate Studies and Research, Alexandria University, Alexandria 21526, Egypt

**Keywords:** wheat straw, *Penicillium chrysogenum* strain A3 DSM105774, xylanase, submerged state of fermentation, low cost eco-friendly methodology

## Abstract

The present work highlights the valorization of the bulky recalcitrant lignocellulose byproduct wheat straw (WS) for the enhanced production of value-added xylanase by the locally sourced novel *Penicillium chrysogenum* strain A3 DSM105774 for the first time. The optimized production of xylanase by submerged state of fermentation of WS was achieved using a three-step statistical and sequential approach: one factor at a time (OFAT), Plackett–Burman design (PBD), and Box Behnken design (BBD). Incubation temperature (30 °C), WS, and ammonium sulphate were the key determinants prompting xylanase production; inferred from OFAT. The WS concentration (%(*w*/*v*)), yeast extract concentration (%(*w*/*v*)), and initial pH of the production medium imposed significant effects (*p* ≤ 0.05) on the produced xylanase, realized from PBD. The predicted levels of WS concentration, initial pH of the production medium, and yeast extract concentration provoking the ultimate xylanase levels (53.7 U/mL) with an 8.95-fold enhancement, localized by the estimated ridge of the steepest ascent of the ridge analysis path, were 3.8% (*w*/*v*), 5.1, and 0.098% (*w*/*v*), respectively; 94.7% lab validation. The current data underpin the up-scaling of xylanase production using this eco-friendly, cheap, and robust methodology for the valorization of WS into the value-added product xylanase.

## 1. Introduction

Millions of tons of agro-industrial waste byproducts do generate from agricultural practices on lignocellulose biomasses annually worldwide. Wheat straw, wheat bran, wheat husk, corncob, rice straw, rice bran, rice husk, oat husk, oat straw, barley straw, sorghum straw, paddy straw, sugarcane straw, and maize straw are paradigms of agro-industrial waste byproducts containing lignocelluloses [1]. 

Wheat straw (designated hereafter as WS) is a recalcitrant agro-industrial waste byproduct containing lignocelluloses. Wheat straw, the dry stalk of wheat after the removal of the grain and the chaff [2], is composed of carbohydrates as a major constituent (27% cellulose, 21% hemicellulose, and 23% lignin on dry weight basis), minerals, proteins, silica, ash, and acid detergent fibers [3,4,5]. The worldwide consumption of wheat was estimated to reach 652.18 million ton in 2010 [6]. Normally, WS is collected and conserved in straw bales. Despite the involvement of WS in an extensive range of applications in medicine [7], fermentation industry [8], soil fertility [9], bio charcoal [10], basket making, pulp industry [11], and bioremediation [12,13], novel usages are urgently mandatory for effective management and economic valorization of the bulky amounts of this byproduct waste. 

Wheat straw is a potential zero-cost alternate for the highly expensive synthetic substrates included in the manufacturing of value-added bioproducts like enzymes by the aid of a panel of filamentous fungi. For instance, *Penicillium janthinellum* [14], 2015), *Trichoderma harzianum* ZF-2 [15], *P. chrysogenum* [16], *Paecilomyces thermophila* J18 [17], *Aspergillus ochraceus* [18], and *A. niger* strain [19] were reported as potential fungi not only for the management of the lignocellulose byproduct WS but also for the WS valorization into value added products in the form of cellulases, laccases, α-amylases, xylanases, and β-xylosoxidases, respectively. 

β-1,4-Xylanases (EC 3.2.1.8) of microbial origin are industrial enzymes with immense biotechnological applications for mankind such as waste treatment, paper-pulp, feed, and food industries [9,20] as well as the manufacturing of biofuel from cellulosic biomass [21]. Over the few last decades, numerous fungal strains were tried for the production of live xylanases using lignocelluloses agro-industrial waste byproducts such as sorghum straw by *Thermomyces lanuginosus* (D_2_W_3_) [22], rice husk by *T. lanuginosus* strain A3-1 DSM 105773 [23], rice bran by *Humicola lanuginosa* [24], rice straw by *Aspergillus fumigatus* NITDGPKA3 [25], wheat bran by *P. chrysogenum* PCL501 [26], wheat straw by *Paecilomyces thermophia* [27] and *Melanocarpus albomyces* IITD3A [28], and maize straw by *Trichoderma viride* [29]. Several *Penicillum* spp (e.g., *Penicillium oxalicum* ZH-30 [30], *P. oxalicum* T3.3 [31], *P. pinophilum* NTG1 II/6 [32], *P. decumbens* [33], *P. janthinelum* CRC 87M-115 [34], *P. purpurogenum* [35], *P. echiulatum* [36], *P. expansum* [37], *P. chrysogenum* PCL501 [26], and *P. canescens* (10-10c) [38] have been intensely studied for their capability to manufacture live xylanases upon their cultivation on wheat bran, wheat straw, rice straw, corncob, oat husk, and barely straw. However, the valorization of WS to a value-added product xylanase by *P. chrysogenum* (recently designated as *P. rubens*) has not been attempted yet. Currently, the xylanase producers at the industrial scale are mostly species of *Aspergillus* and *Trichoderma*. Nonetheless, the search of novel xylanase hyper-producer fungal strains alongside with the selection of zero-cost substrates has captured increasing interest to cover the needs of enzyme markets globally.

In the light of the mentioned above, this work was conducted: (a) to beneficiate the WS through an eco-friendly technology to a valuable end product xylanase, (b) to statistically optimize the xylanase production by the locally novel *Penicillium chrysogenum* strain A3 DSM105774 using WS-based production medium on the flask scale for the first time ever, (b) to customize an inexpensive xylanase production medium containing the zero-cost substrate WS as a co-sole carbon source and xylanase inducer.

## 2. Materials and Methods

### 2.1. Wheat Straw 

The wheat straw waste was obtained from a regional farm in Alexandria City. It was subjected to drying in an oven at 60 °C, and was milled in a Devika commercial mixer grinder, Classic Model, Devika Industries Inc., Gujarat, India) into fluffy flakes (1–2 mm). Then it was preserved in airtight plastic bags at ambient temperature.

### 2.2. Reagents and Chemicals 

Beechwood xylan was obtained from LOBAChemie, PVT., Ltd., Mumbai, India. The 3,5-dinitrosalicylic acid (DNS), xylose was purchased from Sigma-Aldrich Co., Missouri, Saint Louis, USA. The PDA (potato dextrose agar) was purchased from HiMedia Labs, Ltd., Mumbai, India. The18S rRNA fungal-specific primers were manufactured in Macrogen Co., Ltd., Seoul, Korea. 

### 2.3. Media 

The PDA was used for the purposes of activation and short-term maintenance of fungal strains. The minimal medium [39], in g/L: (NH_4_)_2_SO_4_, 1.3; KH_2_PO_4_, 0.37; MgSO_4_·7H_2_O, 0.25; CaCl_2_·2H_2_O, 0.07; FeCl_3_, 0.02; yeast extract 1.0; and 0.5% (*w*/*v*) beechwood xylan, was used as a core xylanase production medium by fungal candidates.

### 2.4. Isolation of Xylanase-Producing Fungal Strains 

The fungal strain enrolled in this work was isolated from a sample gathered from Abbis-2 compost plant, Alexandria City, Egypt. Succinctly, 10 g compost sample was added to 90 mL sterilized saline solution and agitated for 30 min. Ten-fold serial dilution was done followed by plating 0.1 mL of an appropriately selected dilution on PDA plates complemented with streptomycin at a final concentration of 100 mg/L to suppress the growth of bacteria [40]. The incubation temperature for the inoculated PDA plates was 30 °C. After incubation for 6 days, single fungal colonies of distinct colorizations were picked for sub-culturing on PDA plates for further purification. All purified fungal isolates were preserved on PDA slants at 4 °C and sub-cultured monthly. The capability of the tested fungal isolates regarding xylanase production was assessed on a minimal medium complemented with beechwood xylan (0.5% (*w*/*v*)) and 2% (*w*/*v*) agar. The plates were incubated for 6 days at 30 °C followed by overflowing with Congo red (0.1% (*w*/*v*)) for 15 min. The zone of hydrolysis was developed by flooding the plates with 1.5 M NaCl for 30 min. The highest xylanase-producing fungal isolate, selected based on its largest hydrolysis zone surrounding the fungal colony on a xylan agar plate, was selected as the promising fungal strain to conduct this study (Figure S1). 

### 2.5. Scnnaing Electron Microscopy 

The microscopic features of the most promising xylanase-producing fungal strain were investigated under scanning electron microscope (SEM). Dry smears of the fungal strain were coated with approximately 15 nm gold (JEC-1100 E Sputter Coater, JEOL Inc., Pleasanton, CA, USA). After that, the golden coated samples were scanned using SEM (JEOL JSM-5300, JEOL Inc., Pleasanton, CA, USA). The coated slide was accelerated at 30 KV (at room temperature). The digital images of the samples were adjusted and saved for further investigation.

### 2.6. 18S rDNA Sequencing Approach 

Genomic DNA of the quest fungal strain was extracted using the ZR Fungal/Bacterial DNA Miniprep^TM^ (Zymo Co., Irvine, CA, USA) in accordance with the manufacturer’s instructions. The *18S ribosomal DNA* (*18S rDNA*) gene full length of quest fungal strain was amplified by PCR using the fungal specific primer set EF4 (5′-GGAAGGGRTGTATTTATTAG-3′) and EF3 (5′-TCCTCTAAATGACAAGTTTG-3′) [41]. The reaction was conducted at a final volume of 50 µL: 5 µL (30 ng) of genomic DNA, (2.5 µL) 25 pmol of each primer (EF4 and EF3), 25 µL of 1X PCR master mix (iNTRON, Gyeonggi, Korea), and 15 µL nuclease free water. The PCR reaction was executed in PCR thermocycler (Biometra, Göttingen, Germany). PCR conditions were set as follow: initial denaturation at 95 °C for 5 min, 30 cycles (each cycle: denaturation at 94 °C for 45 s, annealing at 48 °C for 45 s and extension at 72 °C for 1.5 min) and final extension at 72 °C for 10 min. The presence of PCR products was checked on 1.0% (*w*/*v*) agarose gel. The 18S rDNA PCR product was purified using the GeneJET PCR Purification kit (Thermo Fisher Scientific Co., Waltham, Massachusetts, MA, USA). Then, the 18S rDNA nucleotide sequence of the fungal strain was searched against the international nucleotide databases (EMBL, GenBank, and DDBJ) using the BLASTN algorithm (http://blast.ncbi.nlm.nih.gov/Blast.cgi, accessed on 15 May 2017). The phylogenetic tree was constructed out via CLC Sequence Viewer 8.0 software to define the evolutionary relationship between the quest fungal isolate and other fungal members. The 18S rDNA nucleotide sequence of the quest fungal strain was deposited in the EMBL database.

### 2.7. Xylanase Assay 

The xylanase activity of the fungal-free supernatant was determined by estimating the reducing sugars derived from the substrate beechwood xylan by the 3, 5 dinitrosalicylic acid (DNS) [42]. Concisely, 0.5 mL beechwood xylan (1.0% (*w*/*v*)) in 100 mL (0.1M sodium acetate buffer, pH 5.0) was added to crude enzyme preparation (0.5 mL) and incubated at 50 °C for 30 min. The reaction was stopped by the addition of DNS reagent (2.0 mL) followed by boiling at 100 °C for 5 min. Unlike the test reaction, the crude enzyme preparation was boiled for 5 min prior to its addition to the reaction mixture in the control reaction. All reaction tubes were cooled down followed by the addition of 7.0 mL distilled water to each tube with thorough mixing. The absorbance of the developed color was estimated spectrophotometrically at 540 nm. The standard curve was established using xylose. All assays were executed in 4 replicates. One arbitrary unit (U) of enzyme activity was described as the quantity of enzyme liberating 1 μmol xylose from the substrate beechwood xylan after one min.

### 2.8. Optimization of Xylanase Production 

A three-step optimizing plan, OFAT, PBD, and BBD, was employed to optimize the xylanase production directed by the most potent xylanase-producing fungal strain under submerged state fermentation.

### 2.9. One Factor at a Time (OFAT) 

The initial selection of the physicochemical determinants affecting the xylanase activity by the most potent xylanase-producing fungal strain was done through OFAT methodology. The theory behind this approach is the alteration of one factor at a time regardless of the likely interactions among the tried factors. In this study, the checked physiochemical factors were wheat straw as a carbon source instead of beechwood xylan, inorganic nitrogen source (i.e., sodium nitrate, ammonium sulphate, ammonium citrate, ammonium chloride, ammonium phosphate, and ammonium nitrate), and incubation temperature. Each inorganic nitrogen source was tested at a final concentration of 1.3 g/L at 35 °C. The following different incubation temperatures 25, 30, 35, and 40 °C were tried out. A 50 mL production medium was dispended in Erlenmeyer flasks (100 mL). Two discs of 4 days activated fungal strain were used to inoculate the Erlenmeyer flask-containing production medium. All cultures were shaken at 120 rpm in an incubator shaker for 6 days. The experimental runs were operated in triplicates.

The proper incubation temperature, deduced from OFAT trials, prompted the maximal levels of xylanase was chosen in order to conduct the subsequent optimization trials. The two independent factors imposing significant consequences on xylanase production deduced from OFAT: wheat straw concentration (% (*w*/*v*)) and ammonium sulphate concentration (% (*w*/*v*)) as well as three independent factors; incubation time, yeast extract concentration (%(*w*/*v*)), and initial pH of the production medium were further studied by PDB; the next step in the optimization strategy.

### 2.10. Plackett–Burman Design (PBD) 

In this study, Plackett–Burman (a full factorial design) [43] was employed in order to evaluate the likely considerable linear impact of the five independent factors: yeast extract concentration (*w*/*v*)), initial pH of the production medium, wheat straw concentration (*w*/*v*)), incubation time (days), and ammonium sulphate concentration (*w*/*v*)) on xylanase production. A matrix of twelve trials, full factorial design, was designed by Minitab 17.3 software as presented in Table 1a. Each factor was coded (−1) and (+1) in two levels low level and (high level, respectively. The possible linear impact enforced by the tried independent factors on the production of xylanase was portrayed through a first-order polynomial equation (Equation (1)).

(1) Y=βo+∑βixi

A fifty mL of the production medium was dispensed in 100 mL Erlenmeyer flasks. All cultures were incubated at 120 rpm in an incubator shaker. The independent factors imposing significant influences on the xylanase production [i.e., initial pH of the production medium, yeast extract concentration (% (*w*/*v*)), and wheat straw concentration (% (*w*/*v*))] were tested further in the final stage in the optimization strategy, BBD. However, the independent factors displaying non-significant concerns on the production of xylanase were used at their initial values in the subsequent experiments. 

### 2.11. Box–Behnken Design (BBD)

The key factors, showing significant influences on the production of xylanase were studied through the BBD [44] to determine the optimum level of each key factor in combination with the maximum level of xylanase. Each factor was coded (−1), (0), and (+1) indicating three levels low level, center level, and high level, respectively in a matrix of fifteen trials (Table 2a). The three studied independent factors were wheat straw concentration (% (*w*/*v*)), initial pH of the production medium, and yeast extract concentration (% (*w*/*v*)). All likely forms of interactions among the studied independent factors triggering significant consequences on the production of xylanase were depicted in Equation (2); a second-order polynomial equation.

(2) $$Y=\beta o+\sum_{i=1}^{k}\beta ixi+\sum_{i=1}^{k}\beta iixixi+\sum_{i=1}^{k-1}\sum_{j=2}^{k}\beta ijxixj+\epsilon$$

All experimental trials had performed in Erlenmeyer flasks (100 mL) containing a 50 mL production medium. All experiments of BBD were incubated in an incubator shaker, with agitation at 120 rpm.

### 2.12. Statistical Analyses and Softwares 

The PBD and BBD used in this study had been designed using the Minitab software 17.3. All regression analyses were performed using Minitab software 17.3. The three-dimensional plots were drawn using the Statistica software 13.1. The RSM package (R Development Core team 2016), Comprehensive R Archive Network (http://CRAN.R-project.org/package=rsm, accessed on 17 March 2016), was used in this study for performing statistical, canonical, and ridge analyses. 

## 3. Results

### 3.1. Penicillium Chrysogenum Strain A3 DSM105774 

**Figure 1:**
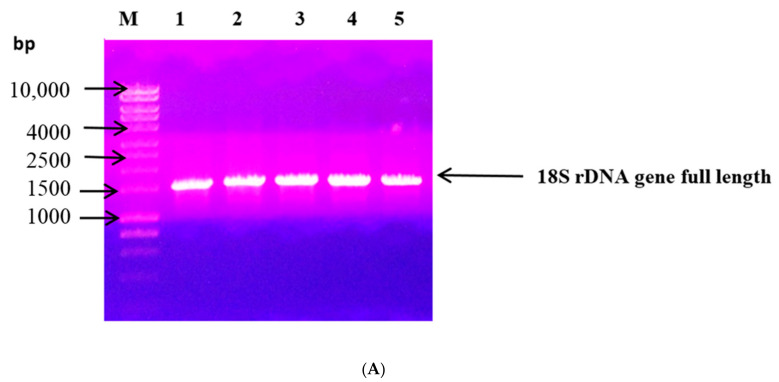

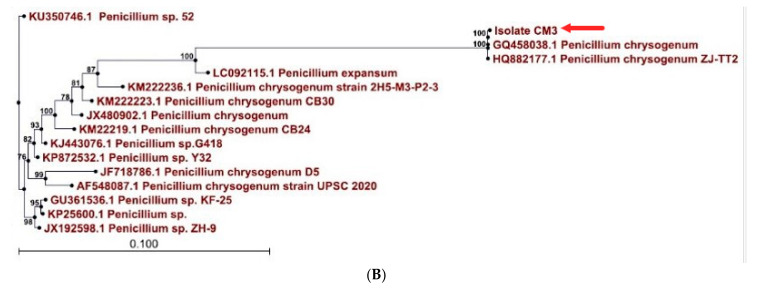
(**A**) Agarose gel (1%*w*/*v*) electrophoresis showing the PCR product for the full length *18S rDNA* gene amplified by PCR from the quest fungal strain. M: 1kbp DNA ladder. Lanes 1–5: amplified PCR product of 18S rDNA full length from the quest fungal strain from five PCR reactions Eppendorf tubes. (**B**) Neighbor-joining tree (constructed by CLC Sequence Viewer 8.0) depicting the phylogenetic relationship between 18S ribosomal DNA sequence of the Figure 3, (the fungal isolate in this study) and other 18S ribosomal DNA sequences assigning to closely related fungi. Bootstrap values were presented on branch nodes (100 re-samplings). The fungal isolate CM3 is the quest fungal strain *P. chrysogenum* strain A3 DSM105774 and is indicated by a red arrow.

**Figure 2:**
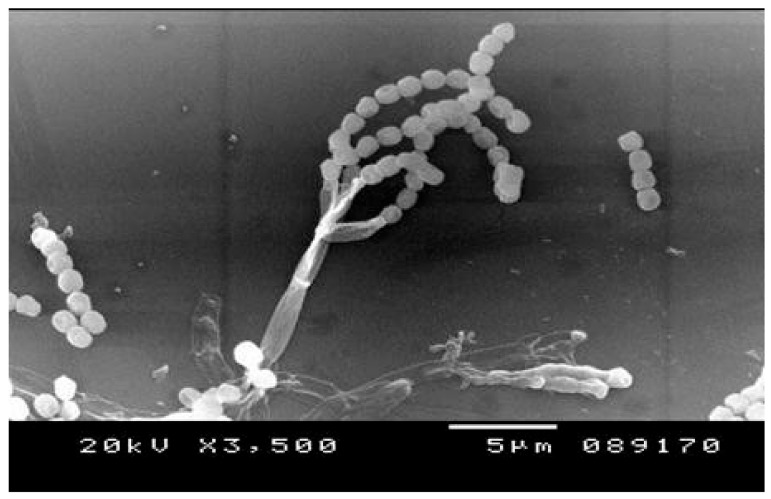
A photo of scanning electron microscope showing the conidia of *P. chrysogenum* A3 DSM105774. The magnification is ×3500.

The full length of the *18S rDNA* gene (1500 bp) of the quest fungal strain was successfully amplified by PCR as depicted in Figure 1A. The analysis of the obtained 18S rDNA nucleotide sequence by BLASTn followed by a phylogenetic tree construction did reveal that the promising xylanase producer isolate affiliated as *Penicillium chrysogenum* (Figure 1B). To help deposit its 18S rRNA (18S rDNA) nucleotide sequence in GenBank, the fungus was assigned a strain nomenclature strain A3. Thus, the fungal isolate was named *Penicillium chrysogenum* strain A3. The deposited 18S rDNA nucleotide sequence was given the accession number KY010602. To guarantee the accessibility of the fungal strain for the public, DSMZ was chosen to deposit the fungal strain with the accession number DSM105774. Moreover, the microscopic features of the fungal strain, mainly the fungal conidia, were depicted in Figure 2 using a scanning electron microscope. 

### 3.2. Key Factors Directing Xylanase Production

**Figure 3:**
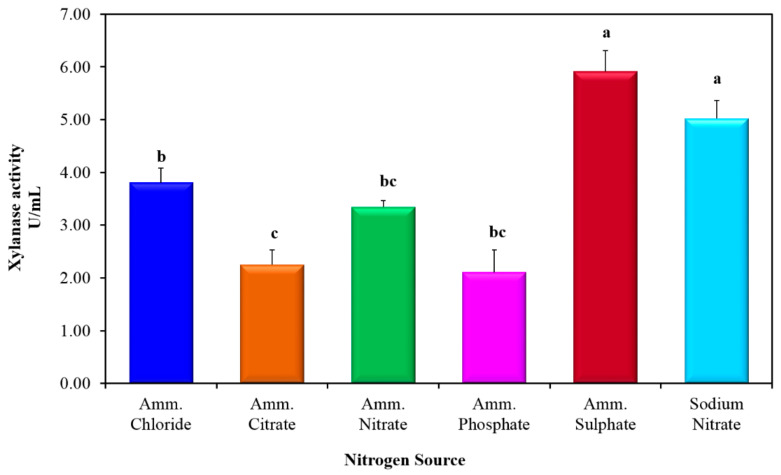
Influence of the nitrogen source on the production of xylanase from *P. chrysogenum* A3 DSM105774 at 35 °C. Values are mean of 4 readings with standard error (SE). Symbols (a, b, and c) displayed on histogram bars were used to distinguish the significance in the xylanase levels imposed by different inorganic nitrogen sources. The histogram bars bearing the same symbol letter indicated non-significant difference. The histogram bars bearing different symbol letters indicated a significant difference at *p*-value ≤ 0.05.

With regard to the influence of nitrogen source on xylanase production via *P. chrysogenum* strain A3 DSM1057, the highest significant levels of xylanase activity (*p* ≤ 0.05) were observed upon the incorporation of ammonium sulphate and sodium nitrate separately in the production medium (Figure 3). No significant difference (*p* ≥ 0.05) in the level of xylanase activity was noticed as a result of the incorporation of ammonium sulphate and sodium nitrate separately in the production medium. Based on the present data, ammonium sulphate was chosen as the appropriate nitrogen source triggering xylanase production in the subsequent experimental trials.

**Figure 4:**
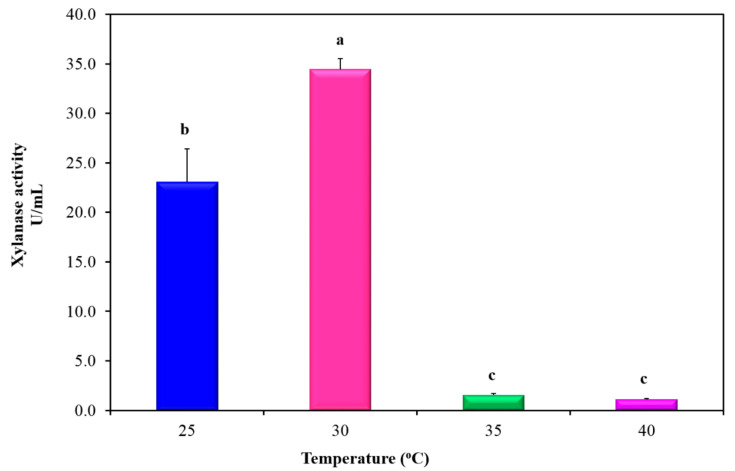
Influence of incubation temperature on the production of xylanase from *P. chrysogenum* A3 DSM105774 using minimal medium complemented with WS (0.5% (*w*/*v*)) for 6 days. Values are mean of 4 readings ± standard errors. Symbols (a, b, and c) displayed on histogram bars were used to distinguish the significance in the xylanase levels imposed by different inorganic nitrogen sources. The histogram bars bearing the same symbol letter indicated non-significant difference. The histogram bars bearing different symbol letters indicated a significant difference at *p*-value ≤ 0.05.

Concerning the impact of the incubation temperature on xylanase production via *P. chrysogenum* strain A3 DSM105774, four incubation temperatures (25, 30, 35 and 40 °C) were studied. Data conferred a dramatic significant reduction (*p* ≤ 0.05) in xylanase activity (1.09637 U/mL) when the fungal strain was cultivated at incubation temperatures higher than 30 °C (i.e., 35 and 40 °C) (Figure 4). Moreover, there was an appreciable variation in xylanase activity (34.4625 and 23.0393 U/mL) (Figure 4) at *p* ≤ 0.05 when the fungus was cultivated at 30 and 25 °C, respectively. Consequently, 30 °C had been chosen as the applicable incubation temperature to carry out the next optimization experiments.

Considering the data derived from OFAT experiments and preceding published reports, the influence of the following key factors: incubation time (days), WS concentration (%(*w*/*v*)), initial pH of the production medium, yeast extract concentration (%(*w*/*v*)), and ammonium sulphate concentration (%(*w*/*v*)) on the process outcome was tested in the next experimental trials of PBD.

### 3.3. Screening of Key Factors Influencing Xylanase Production Using PBD 

The perceivable variations spanning from 4.47 to 15.45 U/mL in xylanase activity (Table 1a), produced by *P. chrysogenum* strain A3 DSM105774, among the twelve empirical runs, evidenced the indispensable need to carry out the optimization plan. Multiple linear regression analysis (Table 1b) evidenced three independent factors: WS concentration (%(*w*/*v*)) (X1), pH of the production medium (X3) and yeast extract concentration (%(*w*/*v*)) (X5) demonstrated improvements (*p* ≤ 0.05) in the levels of xylanase produced by *P. chrysogenum* strain A3 DSM105774. 

The model aptness was evaluated by the co-efficient *R*^2^ that was computed to be 0.866. This *R*^2^-value referred that 86% of the unevenness in the output might be explained by the model. The regression model had multiple co-efficient R-value of 0.93. At most, the best correlation between the predicted and experimental values could be achieved when the R-value is close to 1.0. As a rule of thumb, the regression model with high significance could be inferred from its high F-value (23.3) and low *p*-value (2.8 × 10^−7^). 

The regression coefficients had been calculated with the aid of coded to settle the full polynomial Equation (3) in order to describe the impact of the independent factors on xylanase production by *P. chrysogenum* strain A3 DSM105774.

(3) Y=10.69+3.03X1+0.04X2−0.074X3−0.209 X4−1.25X5

The regression analysis of PDB data inferred that the independent variables (incubation time and ammonium sulphate) did not elicit effects (*p* > 0.05) on the production of xylanase by *P. chrysogenum* strain A3 DSM105774. Consequently, they were settled at their initial levels in the following optimization step. Whilst the independent variables (WS concentration (X1), pH of production medium (X3), and concentration of yeast extract (X5) that exhibited magnitudes (*p* ≤ 0.05) on xylanase production by *P. chrysogenum* strain A3 DSM105774 were assessed in the subsequent step of the optimization plan using BBD.

### 3.4. Pinpointing the Optimal Values of the Key Factors Switching the Production of Xylanase by BBD 

The data of BBD trials for maximizing the production of xylanase by *P. chrysogenum* strain A3 DSM105774 are displayed in Table 2a. The tested three independent variables, derived from the PBD experiment, were WS concentration (%(*w*/*v*)) (X1), pH of the production medium (X3) and yeast extract concentration (%(*w*/*v*)) (X5). The three tried independent factors caused impacts (*p* < 0.05) on xylanase production by *P. chrysogenum* strain A3 DSM105774 through the next formulas of interactions (Table 2b); linear (X1 and X3), quadratic (X1.X1 and X3.X3), and cross form (X1.X3).

Judging of model aptness had been executed through computing co-efficient *R*^2^. The regression model had co-efficient *R*^2^ value of 0.96. This *R*^2^ value deduced that 96% of the unevenness in the outcome might be justified by the model. Moreover, the regression model had a multiple correlation co-efficient *R-*value of 0.98. Normally, the best correlation between the predicted and experimental values could be realized when the *R-* value is close to 1.0. Typically, the high significance of the regression model is attributed to the high F-value and the low *p*-value. Here, the regression model demonstrated F-value (55.86) and *p*-value (3.25 × 10^−12^).

The second-order polynomial Equation (4) had been established with the aid of coded values to depict whole likely types of interactions of the tried independent factors triggering a significant consequence on xylanase production by *P. chrysogenum* strain A3 DSM105774. 


(4) Y=38.45+15.92X1−12.97X3−0.74X5−7.59X1.X1−11.35X3.X3−2.88 X5.X5−8.11X1.X3+0.52X1.X5+1.82X3.X5

**Figure 5:**
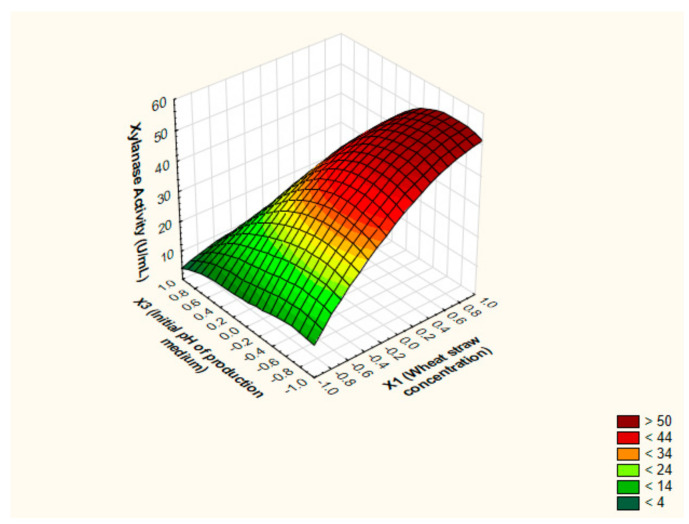
The 3D surface plot depicting the impact of WS concentration (%(*w*/*v*)) and initial pH of the production medium at fixed value of yeast extract concentration (%(*w*/*v*)) on the xylanase production from *P. chrysogenum* A3 DSM105774 in submerged state fermentation.

**Figure 6:**
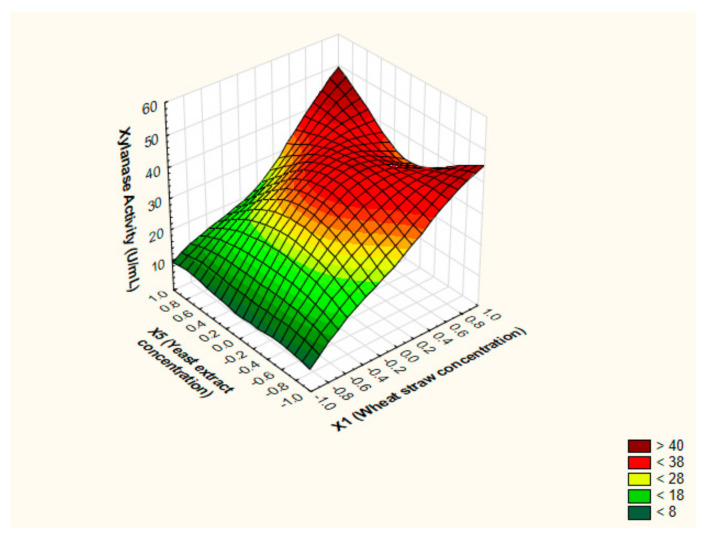
The 3D surface plot depicting the impact of WS concentration (%(*w*/*v*)) and yeast extract concentration (%(*w*/*v*)) at a fixed value of initial pH of the production medium on the xylanase production from *P. chrysogenum* A3 DSM105774 in submerged state fermentation.

**Figure 7:**
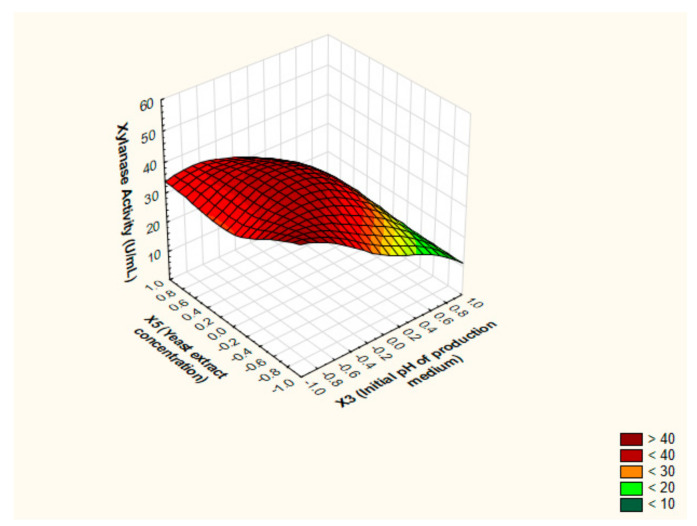
The 3D surface plot depicting the influence of yeast extract concentration (%(*w*/*v*)) and initial pH of the production medium at a fixed value of WS concentration (%(*w*/*v*)) on the production of xylanase from *P. chrysogenum* A3 DSM105774 in submerged state fermentation.

In an attempt to realize the optimized settings for xylanase levels by *P. chrysogenum* strain A3 DSM105774, the characteristics of the response shape was studied by portraying the 3D surface plots. The notion of 3D surface plots relies on investigating the influence of two independent factors simultaneously on output at utmost level of the third independent factor. The predicted stationary points exhibited saddle appearance; as elucidated in Figure 5, Figure 6 and Figure 7. Hence, the ridge and canonical analyses were executed in order to pinpoint the optimum levels of the independent factors and the maximal output parallel. 

### 3.5. Canonical and Ridge Analyses 

The canonical analysis does ascertain the nature of the predicted stationary point; minimum, maximum, or saddle. Eigenvectors in a second-order matrix and eigenvalues could elucidate the form of the response. The surface curvatures could be inferred by eigenvectors [45], whereas the magnitude and the signs of the eigenvalues are an actual signal of the surface form. The notion of the eigenvalues and the mathematical terms were previously clarified [45]. The positive and the negative eigenvalues could indicate responses with upward and downward curvatures, respectively (first rule of Myers). Whilst, the weight of the eigenvalue in its largest value marks off the response curvature in the related direction (second rule of Myers). The present findings prove the eigenvalues: [λ_1_ = −0.0924, λ_3_ = −0.1675 and λ_5_ = −0.4669] for RSM model of xylanase production by *P. chrysogenum* strain A3 DSM105774. The negative eigenvalues of the RSM model revealed the upward curvature of the response in the direction of X5 (yeast extract concentration), with the largest absolute eigenvalue. However, the predicted stationary point for the xylanase activity by *P. chrysogenum* strain A3 DSM105774 was 59.73 U/mL with the predictor set: X1 = 1.679625, X3 = −1.199660, and X3 = −0.358546). Obviously, the predicted stationary point is localized outside the domain. Therefore, ridge analysis is mandatory to obtain further extrapolations for the maximum stationary point. Mostly, the ridge analysis figures the estimated ridge of optimum response from a predictor set at a radius d with steeply growing radii beginning from the origin [46]. Ridge analysis deduced that an increase in the response could happen without realizing the stationary point (threshold level) by moving along the rising ridge. For *P. chrysogenum* strain A3 DSM105774, the predicted rise in the response (xylanase) falling at a distance beyond 1.3 along the rising ridge is unrealistic as all the predictor combination sets would locate outside the explored domain (Table 3). The predictor set: X1 = 1.046, X3 = −0.759, X5 = −0.137 in terms of coded values at a distance of 1.3 inferred the highest, reliable, and estimated response of 56.7 U/mL. By applying the real values of the above-mentioned predictor combination set (3.8% (*w*/*v*), 5.1, and 0.098% (*w*/*v*) for X1 [WS concentration (%(*w*/*v*)], X3 (initial pH of the production medium), and X5 [yeast extract concentration(%(*w*/*v*))], respectively), the laboratory stationary point was 53.7 U/mL; representing 94.7% of the model validation). By the termination of the optimization strategy, the xylanase production by *P. chrysogenum* strain A3 DSM105774 was enhanced 8.95 fold when compared to its level at the beginning of the optimization plan. 

## 4. Discussion

The world xylanase mart is projected to sparkle in the coming years as a result of the continuous and increased obligation of xylanase in several industries. Hence, the continual searching for novel members of live xylanases, produced concomitantly with the most likely lowest expenditures and highest yield, is being addressed to cover the need of worldwide enzyme markets. Meanwhile, the management of the WS is becoming a vital goal to concurrently reduce the possible environmental hazards of this huge recalcitrant lignocellulose byproduct and beneficiate it into a value-added product. 

In the view of the aforementioned, the current work attempted the valorization of WS into the value-added product xylanase by the locally novel *P. chrysogenum* A3 DSM105774 for the first time ever. 

Primarily, the industrialization phase of the xylanase is confined to two prominent aspects: the satisfactory the yield of the elected producer strain and the cost-effectiveness of the bioprocess. In this regard, the indispensable need of searching for a cheap production medium reinforcing xylanase production via *P. chrysogenum* A3 DSM105774 is being obliged. Meanwhile, the whole bioprocess was optimized properly concerning the chemical and the physical key determinants to achieve this goal. The concept of classical methodology for attaining an optimized fermentation process relies on the one factor at a time (OFAT) where one independent factor is studied, and the other factors are fixed at steady levels [47]. This traditional process has some drawbacks mainly time-consuming, lack of precision, discrepancy of results, and unraveled effect of variables interactions. As a consequence, the shortages included in the OFAT approach were covered by employing the statistical sequential PBD and RSM designs. The statistical, sequential, and experimental designs are being broadly employed for the optimization of fermentation factors in the production of enzymes [23,48], antibiotics biodegradation [49], production of microbial pigments [50], production of extracellular pecticoligosaccharides [51], biosynthesis of chitooligosaccharides [52], biodegradation of chicken feather [53], etc. RSM covers a small number of experimental trials with a large number of factors. Additionally, RSM does solve multivariable equations to delimit the optimum levels of key factors regulating the output (response).

These designs were anticipated here in order to determine optimum levels of key factors regulating xylanase yield by *P. chrysogenum* A3 DSM105774. The present finding does indicate that WS can induce considerable levels of xylanase from *P. chrysogenum* A3 DSM105774. The literature of review shows a discrepancy in the appropriate and exploited lignocellulose byproduct which promotes the production of xylanase by an array of fungal strains. For example, wheat bran, persuaded the ultimate xylanase production by *P.chrysogenum* PCL501 [26] and *P. oxalicum* [30]. Whilst, corn cob and sugarcane bagasse did provoke the maximal xylanase production by *P. purpurogenum* [35] and *P. chrysogenum* F-15 [54], respectively. As a rule of thumb, the incorporation of zero-cost substrates (e.g., lignocellulose byproduct) in the production media will definitely decrease the expenditure of the bioprocess. 

Among the physical independent variables, the incubation temperature is a major key determinant, controlling the fermentation processes [55]. Mostly, the optimal incubation temperature prompting the maximal xylanase production by the mesophilic fungi was reported to localize at 25–30 °C. The maximal xylanase production by *P. citrinum* xym2 [56], *P. chrysogenum* F-15 strain [54], and *P. glabrum* [57] was realized at 30, 20, and 25 °C, respectively. On the contrary, the maximum xylanase production by *P. purpurogenum* was accomplished at 40 °C [35]. At most, applying mild incubation temperatures (25–30 °C) in enzyme bioprocessing, would not only prevent the protein misfolding and its undesirable consequences [58] but also contribute greatly to energy saving, accompanying by lower bioprocesses’ expenditures. Nevertheless, applying mild incubation temperatures for production in bioprocesses might increase the likelihood of exposure to bacterial contamination. This would in turn address the indispensable need for applying strict safety precaution protocols during the midstream processing to avoid bacterial contamination and its undesired consequences. 

It is worth mentioning that the type of nitrogen source incorporated in the production medium is one of the key factors regulating the productivity of xylanases from the producers [33]. Unlike *P. chrysogenum* A3 DSM105774 with persuaded xylanase levels in the presence of ammonium sulphate, enhanced xylanase levels by *P. citrinum* xym2 [56] and *Penicillium* sp. AKB-24 [59] were achieved in the presence of di-ammonium hydrogen phosphate and urea (dosing of 0.08% and 0.12%), respectively. Likewise, *P. chrysogenum* A3 DSM105774 with prompted xylanase levels in the presence of yeast extract, significant positive consequences on the xylanase levels from *P. citrinum* isolate HZN13 [60] and *Penicillium* sp.WX-Z1 [61] were imposed by yeast extract.

The incubation time is a key determinant in the schedule of industrialized bioprocessing. Normally, the lengthening of a bioprocess period will increase the total bioprocess expenditure. This in turn focused on the obliged determination of the optimum mandatory period for xylanase synthesis by the fungal strain being studied. Enzyme bioprocessing directed by fungal members is somehow a lengthy process with a time spanning from four to fifteen days when compared to those directed by bacterial members. The optimal time encouraging xylanase production in combination with the maximum levels of xylanase by some reported fungal candidates was four days with 123.1 U/mL from *Penicillium* sp. [62], 5 days with 1.35 Units /mL from *P. chrysogenum* PCL501 [26], six days with 14.50 U/mL from *P. oxalicum* ZH-30 [30], eight days with 0.834 U/mL from *T. lanuginosus* A3-1 DSM 105773 [23], and twelve days with 1.42 U/mL from *Trametes versicolor* [63]. The compulsory time for xylanase production from *P. chrysogenum* A3 DSM105774 is localized within the abovementioned time span as five days.

The pH of the production medium could impose a significant influence on the magnitude value of the xylanase produced by the employed fungal strain. Mostly, the optimum pH prompting xylanase production from acidophiles and neutrophilies fungal candidates are localized in the strong acidic to a slightly neutral range of pH (3.0–7.7). The review of literature stated that the optimal initial pH of the production medium encouraging high xylanase values were pH 6.0 by *P. chrysogenum* F-15 [54], pH 7.72 by *P. oxalicum* ZH-30 [30], pH 5.5 by *P. glabrum* [57], pH 4.0 by *P.citrinum* isolate HZN13 [60], and pH 3.0 by *P. purpurogenum* [35]. *P. chrysogenum* strain A3 DSM105774 did exhibit almost a quite similar trend of an initial pH of the production medium of 5.1, encouraging high xylanase levels. The divergence in the initial pH of the production medium encouraging fungal xylanase production might be likely ascribed to the mechanism exploring the synthesis of xylanase by these disparate fungal strains. Additionally, the preferential initial pH of the production medium for fungal xylanase production might be a consequence of the pH of their natural habitats.

Bioprocess yield is a key element in the schedule of enzyme manufacturing to ensure bioprocess with satisfactory profits. A remarkable fold enhancement (8.95) in the level of xylanase produced by *P. chrysogenum* A3 DSM105774 under study was noticed by the end of the optimization plan. On the other hand, the level of xylanase from *T. lanuginosus* A3-1 DSM105773 was remarkably furthered by a fold improvement of 5.0 [23] and the xylanase yield by *P. purpurogenum* with heightened circumstances was 65.72% greater equaled to traditional conditions [35]. This fold of enhancement would confer not only the adequacy and precision of the applied statistical designs but also the indispensable need to perform the optimization step on the bench scale prior to the transfer to the industrialization step.

Comprehensively, there is an obvious discrepancy among different fungal xylanase producers regarding several issues; of most the importance the ultimate xylanase yield achieved by the employed fungal strain, duration time required for the completion of the bioprocess with the highest probable xylanase yield, type and concentration of each constituent in the production medium, and the type of the lignocellulose byproduct substrate provoking the highest possible xylanase yield. The underlying reasons behind all these discrepancy issues could be outlined in the following arguments [23]: (a) the potential of the producer fungal strain secreting the target enzyme and the co- helper hydrolytic enzyme cascades, (b) the type of substrate in terms of the lignocellulose byproduct, (c) the accessibility of the lignocellulose byproduct substrate to the producer fungal strain, (d) the chemical composition of the lignocellulose byproduct substrate, (e) the approach of the applied fermentation strategy either solid-state or submerged state fermentation, (f) the cultural conditions (e.g., the incubation temperature, the agitation speed, initial pH of the production medium, etc.), (g) the conditions of enzyme assay, (h) the definition of enzyme units, (i) the length of the fermentation process, (j) and the non-carbon/non-nitrogen constituents of the production medium acting as enzyme co-factors.

## 5. Conclusions

In conclusion, the locally isolated *Penicillium chrysogenum* strain A3 DSM105774 was successfully employed in the valorization of the accumulated lignocellulose byproduct WS to a value-added product xylanase with a fold enhancement of 8.95 compared to its level prior to the optimization strategy. Prospective studies will mainly focus on the feasibility of scaling up the current bioprocess using the pilot scale.

## Figures and Tables

**Table 1:** (**a**) Coded-real values of five factors in PBD for observing top significant factors regulating the production of xylanase by *P. chrysogenum* strain A3 DSM105774. (**b**) An outline for multiple linear regression of PBD data for assessment of the production of xylanase by *P. chrysogenum* strain A3 DSM105774.

**(a)**							
**Independent Factors**						**Y** **Xylanase Activity (U/mL)**	
**Trial #**	**X1**	**X2**	**X3**	**X4**	**X5**	**Exp. ^a^**	**Pred. ^b^**
1	1(1.0)	−1(0.065)	1(7.0)	−1(4.0)	−1(0.025)	14.43	14.39
2	1(1.0)	1(0.25)	−1(5.0)	1(8.0)	−1(0.025)	15.00	15.54
3	−1(0.25)	1(0.25)	1(7.0)	−1(4.0)	1(0.15)	6.54	5.92
4	1(1.0)	−1(0.065)	1(7.0)	1(8.0)	−1(0.025)	13.47	13.98
5	1(1.0)	1(0.25)	−1(5.0)	1(8.0)	1(0.15)	14.32	13.04
6	1(1.0)	1(0.25)	1(7.0)	−1(4.0)	1(0.15)	9.63	11.98
7	−1(0.25)	1(0.25)	1(7.0)	1(8.0)	−1(0.025)	9.39	7.99
8	−1(0.25)	−1(0.065)	1(7.0)	1(8.0)	1(0.15)	6.21	5.42
9	−1(0.25)	−1(0.065)	−1(5.0)	1(8.0)	1(0.15)	4.47	6.89
10	1(1.0)	−1(0.065)	−1(5.0)	−1(4.0)	1(0.15)	15.45	13.38
11	−1(0.25)	1(0.25)	−1(5.0)	−1(4.0)	−1(0.025)	9.48	9.89
12	−1(0.25)	−1(0.065)	−1(5.0)	−1(4.0)	−1(0.025)	9.84	9.81
**(b)**							
**Independent Factor**	**Estimate Symbol**	**Estimate**	**t-Value**	***p*-Value**		**Confidence Level (%)**	
Intercept	B°	10.68575	34.2592	7.64 × 10^−18^		100.00 *	
X1	B1	3.030083	9.714641	1.39 × 10^−8^		99.99 *	
X2	B2	0.040083	0.12851	0.899171			
X3	B3	−0.74008	−2.37275	0.029001		97.10 *	
X4	B4	−0.20925	−0.67087	0.510822			
X5	B5	−1.24925	−4.00518	0.000830		99.91 *	

Values between brackets: real values for the independent factors. X1: WS concentration (%(*w*/*v*)), X2: ammonium sulphate concentration (%(*w*/*v*)), X3: initial pH of the production medium, X4: incubation time (days) and X5: yeast extract concentration (%(*w*/*v*)). ^a^: experimental values and ^b^: predicted values. Values: mean of 3 readings. * Significant *p*-value: ≤ 0.05.

**Table 2:** (**a**) The levels of coded and real values of three independent factors in BBD for maximizing the xylanase production by *P. chrysogenum* strain A3 DSM105774. (**b**) Regression synopsis of BBD for assessing xylanase levels produced by *P. chrysogenum* strain A3 DSM105774.

**(a)**					
**Trial #**	**X1**	**X3**	**X5**	**Y6** **Xylanase Activity (U/mL)**	
				**Exp. ^a^**	**Pred. ^b^**
1	−1(0.1)	−1(4.5)	0(0.056)	10.77	8.45
2	1(1.9)	−1(4.5)	0(0.056)	51.42	56.52
3	−1(0.1)	1(9.5)	0(0.056)	3.84	−1.26
4	1(1.9)	1(9.5)	0(0.056)	12.03	14.35
5	−1(0.1)	0(7.0)	−1(0.012)	7.26	13.32
6	1(1.9)	0(7.0)	−1(0.012)	45.48	44.13
7	−1(0.1)	0(7.0)	1(0.1)	9.45	10.80
8	1(1.9)	0(7.0)	1(0.1)	49.74	43.68
9	0(1.0)	−1(4.5)	−1(0.012)	43.50	39.75
10	0(1.0)	1(9.5)	−1(0.012)	11.13	10.17
11	0(1.0)	−1(4.5)	1(0.1)	33.66	34.62
12	0(1.0)	1(9.5)	1(0.1)	8.58	12.33
13	0(1.0)	0(7.0)	0(0.56)	37.47	38.45
14	0(1.0)	0(7.0)	0(0.56)	38.82	38.45
15	0(1.0)	0(7.0)	0(0.56)	39.06	38.45
**(b)**					
**Independent Factor**	**Estimate Symbol**	**Estimate**	**t-Value**	***p*-Value**	**Confidence Level (%)**
Intercept	B°	38.45	22.75182	9.1 × 10^−6^	100.00 *
X1	B1	15.92	15.38201	1.51 × 10^−12^	99.99 *
X3	B2	−12.97	−12.5339	6.28 × 10^−11^	99.999 *
X5	B3	−0.74	−0.71746	0.481384	-
X1.X1	B11	−7.59	−4.97925	7.21 × 10^−5^	99.99 *
X3.X3	B22	−11.35	−7.45082	3.44 × 10^−7^	99.999 *
X5.X5	B33	−2.88	−1.89225	0.073025	-
X1.X3	B12	−8.11	−5.5447	1.99 × 10^−5^	99.99 *
X1.X5	B13	0.52	0.35359	0.727346	-
X3.X5	B23	1.82	1.245251	0.227433	-

Values between brackets: real values for the independent factors. X1: WS concentration (%(*w*/*v*)), X3: initial pH of the production medium and X5: yeast extract concentration (%(*w*/*v*)). ^a^: experimental values and ^b^: predicted values. Values: mean of 3 readings.* Significant *p*-values: ≤ 0.05.

**Table 3:** The estimated ridge of xylanase activity produced by *P. chrysogenum* strain A3 DSM105774 through the steepest ascent path of ridge analysis.

Distance (d)	Independent Factor *			Xylanase Activity (U/mL)
	X1	X3	X5	
0.0	0.000	0.000	0.000	38.46
0.1	0.078	−0.062	−0.004	40.44
0.3	0.237	−0.184	−1.093	44.16
0.5	0.397	−0.184	−0.030	47.46
0.7	0.559	−0.184	−0.049	50.37
0.9	0.722	−0.533	−0.072	52.86
1.1	0.884	−0.647	−0.101	54.99
1.3 **	1.046	−0.759	−0.137	56.7
1.5	1.208	−0.871	−0.179	58.05
1.7	1.368	−0.982	−0.230	58.98
1.9	1.527	−1.093	−0.290	59.55
0.0	0.000	0.000	0.000	38.46
0.1	0.078	−0.062	−0.004	40.44

* Independent factors in terms of coded values. ** The predictor combination set exhibiting the highest yield of xylanase inferred from the steepest ascent path of ridge analysis. X1: WS concentration (%(*w*/*v*)), X3: initial pH of the production medium, and X5: yeast extract concentration (%(*w*/*v*)).

## Supplementary Materials

The following are available online at https://www.mdpi.com/article/10.3390/jof7090696/s1, Figure S1: Representative screening of xylanase-producing fungi on beechwood xylan-containing agar plate showing a clear hydrolytic zone around the fungal colony against red background after flooding the plate with 0.1%(*w*/*v*) Congo red.

## References

[1] Anwar Z., Gulfraz M., Irshad M. (2014). Agro-industrial lignocellulosic biomass a key to unlock the future bio-energy: A brief review. J. Radiat. Res. Appl. Sc..

[2] Gubitz G.M., Mansfield S.D., Bohm D., Saddler J.N. (1998). Effect of endoglucanases and hemicelluloses in magnetic and floatation deinking of xerographic and laser printed papers. J. Biotechnol..

[3] McKendry P. (2002). Energy production from biomass (part 1): Overview of biomass. Bioresour. Technol..

[4] Yasin M., Bhutto A.W., Bazmi A.A., Karim S. (2010). Efficient utilization of rice-wheat straw to produce value added composite products. Int. J. Chem. Env. Eng..

[5] Adapa P.K., Tabil L.G., Schoenau G.J., Canam T., Dumonceaux T. (2011). Quantitative analysis of lignocellulosic components of non-treated and steam exploded barley, canola, oat and wheat straw using fourier transform infrared spectroscopy. J. Agric. Sci. Technol..

[6] WASDE (2010). World Agricultural Supply and Demand Estimates, 2010.

[7] Drankham K., Carter J., Madl R., Klopfenstein C., Padula F., Lu Y., Warren T., Schmitz N., Takemoto D. (2003). Antitumor activity of wheats with high orthophenolic content. Nutr. Cancer.

[8] Mojsov K. (2010). Application of solid state fermentation for cellulase enzyme production using *Trichoderma viride*. Perspect. Innov. Econ. Bus..

[9] Murray T.D., Bruehl G.W. (1983). Composition of wheat straw infested with *Cephalosporium gramineum* and implications for its decomposition in soil. Phytopathology.

[10] Goodall C. (2010). Biochar: Ten Technologies to Save the Planet.

[11] CWC and Domtar Inc. (1997). Wheat Straw as a Fiber Source: Recycling Technology Assistance Partnership (RTAP).

[12] Dupont L., Bounanda J., Domonceau J., Aplincourt M. (2003). Metal ions binding onto a lignocellulosic substrate extracted from wheat bran: A NICA-donnon approach. J. Colloid Interface Sci..

[13] Al-Kindi S., Abed R.M. (2016). Effect of Biostimulation using sewage sludge, soybean meal, and wheat straw on oil degradation and bacterial community composition in a contaminated desert soil. Front. Microbiol..

[14] Sharma B., Agrawal R., Singhania R.R., Satlewal A., Mathur A., Tuli D., Adsul M. (2015). Untreated wheat straw: Potential source for diverse cellulolytic enzyme secretion by *Penicillium janthinellum* EMS-UV-8 mutant. Biores. Tech..

[15] Gao H., Chu X., Wang Y., Zhou F., Zhao K., Mu Z., Liu Q. (2013). Media optimization for laccase production by *Trichoderma harzianum* ZF-2 using response surface methodology. J. Microbiol. Biotechnol..

[16] Balkan B., Ertan F. (2007). Production of alpha amylase from *Penicillium chrysogenum* under solid-state fermentation by using some agricultural by-products. Food Technol. Biotechnol..

[17] Yang S.Q., Yan Q.J., Jiang Z.Q., Li L.T., Tian H.M., Wang Y.Z. (2006). High-level of xylanase production by the thermophilic *Paecilomyces themophila* J18 on wheat straw in solid-state fermentation. Biores. Technol..

[18] Michelin M., Maria de Lourdes T.M., Ruzene D.S., Silva D.P., Vicente A.A., Jorge J.A., Terenzi H.F., Teixeira J.A. (2012). Xylanase and β-xylosidase Production by *Aspergillus ochraceus*: New perspectives for the application of wheat straw autohydrolysis liquor. Appl. Biochem. Biotechnol..

[19] Azzouz Z., Bettache A., Boucherba N., Prieto A., Martinez M.J., Benallaoua S., de Eugenio L.I. (2021). Optimization of β-1,4-endoxylanase production by an *Aspergillus niger* strain growing on wheat straw and application in xylooligosaccharides production. Molecules.

[20] Ninawe S., Kuhad R.C. (2006). Bleaching of wheat straw-rich soda pulp with xylanase from a thermoalkalophilic *Streptomyces cyaneus* SN32. Biores. Technol..

[21] Ali U.F., El-Dein H.S.S. (2008). Production and partial purification of cellulase complex by *Aspergillus niger* and *A. nidulans* grown on water hyacinth blend. J. Appl. Sci. Res..

[22] Sonia K.G., Chadha B.S., Saini H.S. (2005). Sorghum straw for xylanase hyper-production by *Thermomyces lianuginosus* (D2W3) under solid-state fermentation. Biores. Technol..

[23] Matrawy A.A., Khalil A.I., Marey H.S., Embaby A.M. (2020). Biovalorization of the raw agro-industrial waste rice husk through directed production of xylanase by *Thermomyces lanuginosus* strain A3-1 DSM 105773: A statistical sequential model. Biomass Conv. Bioref..

[24] Rajoka M.I., Huma T., Khalid A.M., Latif F. (2005). Kinetics of enhanced substrate consumption and endo-β-xylanase production by a mutant derivative of *Humicola lanuginosa* in solid-state fermentation. World J. Microbiol. Biotechnol..

[25] Sarkar N., Aikat K. (2012). Cellulose and xylanase production from rice straw by a locally isolated fungus *Aspergillus fumigatus* NITDGPKA3 under solid state fermentation-statistical optimization by response surface methodology. J. Technol. Innov. Renew. Energy.

[26] Okafor U.A., Emezue T.N., Okochi V.I., Onyegeme-Okerenta B.M., NwodoChinedu S. (2007). Xylanase production by *Penicillium chrysogenum* (PCL501) fermented on cellulosic wastes. Afr. J. Biochem. Res..

[27] Yang S., Wang Y., Jiang Z., Hua C. (2008). Crystallization and preliminary X-ray analysis of a 1,3–1,4-β-glucanase from *Paecilomyces thermophile*. Acta Crystallogr. Sect. F Struct. Biol. Cryst. Commun..

[28] Biswas R., Sahai V., Mishra S., Bisaria V.S. (2010). Bioprocess strategies for enhanced production of xylanase by *Melanocarpus albomyces* IITD3A on agro-residual extract. J. Biosci. Bioeng..

[29] Goyal M., Kalra E.L., Sarren V.K., Soni G. (2008). Xylanase production with xylan rich lignocellulosic wastes by a local soil isolate of *Trichoderma viride*. Braz. J. Microbiol..

[30] Yin L., Fengjie C., Zhiqiang L., Yingying X., Hui Z. (2007). Improvement of xylanase production by *Penicillium oxalicum* ZH-30 using response surface methodology. Enzym. Microb. Technol..

[31] Zahari N.I., Shah U.K.M., Asa’ari A.Z.M., Mohamad R. (2016). Selection of potential fungi for production of cellulase-poor xylanase from rice straw. Bioresources.

[32] Brown J.A., Collin S.A., Wood T.M. (1987). Development of a medium for high cellulase, xylanase and fl-glucosidase production by a mutant strain (NTG III/6) of the cellulolytic fungus *Penicillium pinophilum*. Enzym. Microbl. Technol..

[33] Sun X., Zhang R., Zhang Y. (2004). Production of lignocellulolytic enzymes by *Trametes gallica* and detection of polysaccharide hydrolase and laccase activities in polyacrylamide gels. J. Basic Microbiol..

[34] Oliveira L., Porto A., Tambourgi E. (2006). Production of Xylanase and Protease by *Penicillium janthinellum* CRC 87M-115 from Different Agricultural Wastes. Bioresour. Technol..

[35] Sunkar B., Kannoju B., Bhukya B. (2020). Optimized production of xylanase by *Penicillium purpurogenum* and ultrasound impact on enzyme kinetics for the production of monomeric sugars from pretreated corn cobs. Front. Microbiol..

[36] Camassola M., Dillon A.J.J. (2007). Production of cellulases and hemicellulases by *Penicillium echinulatum* grown on pretreated sugar cane bagasse and wheat bran in solid-state fermentation. Appl. Microbiol..

[37] Querido A., Coelho J., Araújo E., Chaves-Alves V. (2006). Partial purification and characterization of xylanase produced by *Penicillium expansum*. Braz. Arch. Biol. Technol..

[38] Bakri Y., Jacques P., Thonart P. (2003). Xylanase production by *Penicillium canescens* 10-10c in Solid-state fermentation. Appl. Biochem. Biotechnol..

[39] Fusawat P., Rakariyatham N. (2014). Potential of cellulase and xylanase production by fungal strains using corn husks as substrate. Asia Pac. J. Sci. Technol..

[40] Adeleke M.A., Akatah H.A., Hassan A.O., Adebimpe W.O. (2012). Microbial load and multiple drug resistance of pathogenic bacteria isolated from feaces and body surfaces of cockroaches in an urban area of southwestern Nigeria. J. Microbiol. Biotechnol. Food Sci..

[41] Smit E., Leeflang P., Glandorf B., Van Elsas J.D., Wernars K. (1999). Analysis of fungal diversity in the wheat rhizosphere by sequencing of cloned PCR-amplified genes encoding *18S rRNA* and temperature gradient gel electrophoresis. Appl. Environ. Microbiol..

[42] Miller G.L. (1959). Use of dinitrosalicylic acid reagent for determination of reducing sugar. Anal. Chem..

[43] Plackett R.L., Burman J.P. (1946). The design of optimum multifactorial experiments. Biometrika.

[44] Box G.E., Behnken D.W. (1960). Some new three level designs for the study of quantitative variables. Technometrics.

[45] Myers R.H. (1976). Response Surface Methodology.

[46] Draper N.R. (1963). Ridge analysis of response surfaces. Technometrics.

[47] Khucharoenphaisan K., Tokuyama S., Kitpreechavanich V. (2008). Statistical optimization of activity and stability of β-xylanase produced by newly isolated *Thermomyces lanuginosus* THKU-49 using central composite design. Afr. J. Biotechnol..

[48] Embaby A.M., Masoud A.A., Marey H.S., Shaban N.Z., Ghonaim T.M. (2014). Raw agro-industrial orange peel waste as a low cost effective inducer for alkaline polygalacturonase production from *Bacillus licheniformis* SHG10. SpringerPlus.

[49] Anan A., Ghanem K.M., Embaby A.M., Hussein A., El-Naggar M.Y. (2018). Statistically optimized ceftriaxone sodium biotransformation through *Achromobacter xylosoxidans* strain Cef6: Unusual insight for bioremediation. J. Basic Microbiol..

[50] Embaby A.M., Hussein M.N., Hussein A. (2018). Monascus orange and red pigments production by *Monascus purpureus* ATCC16436 through co-solid state fermentation of corn cob and glycerol: An eco-friendly environmental low cost approach. PLoS ONE.

[51] Embaby A.M., Melika R.R., Hussein A., El-Kamel A., Marey H.S. (2016). A Novel non-cumbersome approach towards biosynthesis of pectic-oligosaccharides by non-aflatoxigenic *Aspergillus* sp. Section Flavi Strain EGY1 DSM 101520 through citrus pectin fermentation. PLoS ONE.

[52] Embaby A.M., Melika R.R., Hussein A., El-Kamel A.H., Marey H.S. (2018). Biosynthesis of chitosan-oligosaccharides (COS) by non-aflatoxigenic *Aspergillus* sp. strain EGY1 DSM 101520: A robust biotechnological approach. Process. Biochem..

[53] Embaby A.M., Marey H.S., Hussein A. (2015). A statistical-mathematical model to optimize chicken feather waste bioconversion via *Bacillus licheniformis* SHG10: A low cost effective and ecologically safe approach. J. Bioprocess Biotech..

[54] Terrone C., Freitas C., Fanchini Terrasan C.R., Almeida A.L., Carmona E. (2018). Agroindustrial biomass for xylanase production by *Penicillium chrysogenum*: Purification, biochemical properties, and hydrolysis of hemicelluloses. Electron. J. Biotechnol..

[55] Krishna C. (2005). Solid-state fermentation systems—An overview. Crit. Rev. Biotechnol..

[56] Saha S.P., Ghosh S. (2014). Optimization of xylanase production by *Penicillium citrinum* xym2 and application in saccharification of agro-residues. Biocatal. Agric. Biotechnol..

[57] Knob A., Beitel S.M., Fortkamp D., Terrasan C.R.F., Almeida A.F.D. (2013). Production, purification, and characterization of a major *Penicillium glabrum* xylanase using brewer’s spent grain as substrate. BioMed Res. Int..

[58] Mogk A., Mayer M.P., Deuerling E. (2002). Mechanisms of protein folding: Molecular chaperones and their application in biotechnology. Chembiochem.

[59] Kumar A., Gautam A., Dutt D. (2016). Co-Cultivation of *Penicillium* sp. AKB-24 and *Aspergillus nidulans* AKB-25 as a cost-effective method to produce cellulases for the hydrolysis of pearl millet stover. Fermentation.

[60] Bagewadi Z.K., Mulla S.I., Shouche Y., Ninnekar H.Z. (2016). Xylanase production from *Penicillium citrinum* isolate HZN13 using response surface methodology and characterization of immobilized xylanase on glutaraldehyde-activated calcium-alginate beads. 3 Biotech.

[61] Cui F., Zhao L. (2012). Optimization of xylanase production from *Penicillium* sp. WX-Z1 by a two-step statistical strategy: Plackett-Burman and Box-Behnken experimental design. Int. J. Mol. Sci..

[62] Sridevi A., Golla N., Suvarnalatha P. (2019). Production of xylanase by *Penicillium* sp. and its biobleaching efficiency in paper and pulp industry. Int. J. Pharm. Sci. Res..

[63] Khalil. A.I., Krakowiak A., Russel S. (2002). Production of extracellular cellulase and xylanase by the ligninolytic white-rot fungus *Trametes versicolor* grown on agricultural wastes. Ann. Agric. Sci..

