# Supplementary material for: Use of Wheat Straw for Value-Added Product Xylanase by Penicillium chrysogenum Strain A3 DSM105774
Source: J Fungi (Basel). 2021 Aug 27;7(9):696. doi: 10.3390/jof7090696 (PMC8472069; doi:10.3390/jof7090696)
Supplement: Supplementary file 1 [file jof-07-00696-s001.zip › jof-1320339-supplementary.pdf]

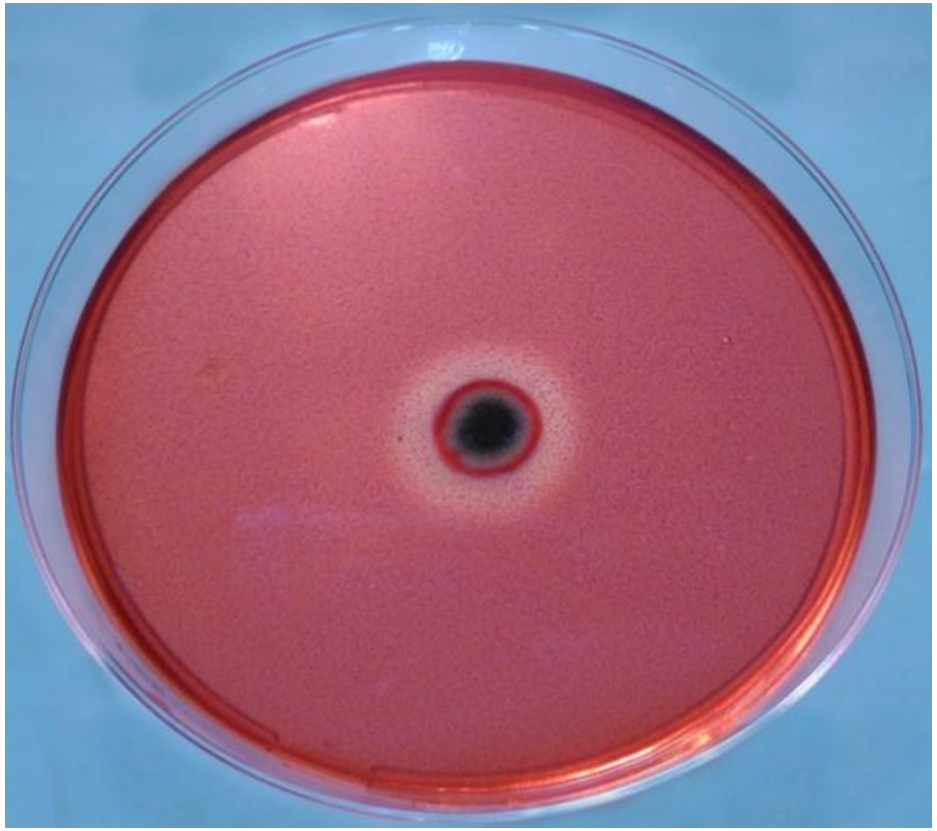

Figure S1. Representative screening of xylanase-producing fungi on beechwood xylan-containing agar plate showing a clear hydrolytic zone around the fungal colony against red background after flooding the plate with 0.1%(w/v) Congo red.
